# Development of a Rapid *in planta* BioID System as a Probe for Plasma Membrane-Associated Immunity Proteins

**DOI:** 10.3389/fpls.2018.01882

**Published:** 2018-12-18

**Authors:** Brendon Conlan, Thomas Stoll, Jeffrey J. Gorman, Isabel Saur, John P. Rathjen

**Affiliations:** ^1^Research School of Biology, The Australian National University, Acton, ACT, Australia; ^2^QIMR Berghofer Medical Research Institute, Herston, QLD, Australia

**Keywords:** pathogen, immunity, plasma membrane, BioID, AvrPto, plant, effector, biotin

## Abstract

Plant pathogens secrete effector molecules that suppress the plant immune response to facilitate disease development. AvrPto is a well-studied effector from the phytopathogenic bacterium *Pseudomonas syringae*. Here we utilize an *in planta* proximity dependent biotin ligase labeling technique (BioID) in combination with AvrPto to identify proximal proteins that are potential immune system components. The labeling technique biotinylated proteins proximal to AvrPto at the plasma-membrane allowing their isolation and identification by mass spectrometry. Five AvrPto proximal plant proteins (APPs) were identified and their effect on plant immune function and growth was examined in *Nicotiana benthamiana* leaves. One protein identified, RIN4, is a central immune component previously shown to interact with AvrPto. Two other proteins were identified which form a complex and when silenced significantly reduced *P. syringae tabaci* growth. The first was a receptor like protein kinase (APK1) which was required for Pto/Prf signaling and the second was Target of Myb1 (TOM1), a membrane associated protein with a phosphatidylinositol 5-phosphate (PtdIns5P) binding motif. We have developed a technology to rapidly determine protein interactions within living plant tissue. It is particularly useful for identifying plant immune system components by defining pathogenic effector protein interactions within their plant hosts.

## Introduction

Plants employ receptor kinases (RKs) and receptor proteins as pattern recognition receptors (PRRs) to sense microbes. To counter plant defense mechanisms, pathogens utilize cytoplasm targeted effector proteins to alter plant cell function and metabolism to aid infection. Many bacterial effectors target PRRs and their signaling components to prevent immune recognition of the pathogen (Jones and Dangl, 2006). Progress into deciphering how effector proteins elicit their response on the plant immune system is being made (Dodds and Rathjen, 2010; Deslandes and Rivas, 2012), with recent evidence indicating effectors converge on central hubs in the plant immune system network through effector redundancy (Mukhtar et al., 2011; Weßling et al., 2014). Effector proteins are useful tools for dissecting plant biology due to their abilities to target key pathways in the host cell. A significant portion of these effector interactions occur at the plasma membrane interface, where the biophysical environment changes due to restriction from three-dimensional space to two dimensions. Proteins anchored in a membrane may display strong interactions but when the cell is homogenized and the membrane is no longer acting to stabilize the interface then the interaction may be lost. As a result, there are likely many interactions that are overlooked by traditional protein–protein interaction screens. Understanding the spectrum of plant immunity components influenced by effector protein interactions is paramount to identifying solutions for engineering plant disease resistance.

The effector AvrPto from the tomato pathogen *Pseudomonas syringae* pv. *tomato (Pst)* is known to interact with a number of proteins associated with plant immunity (Scofield et al., 1996; Xiang et al., 2008; Luo et al., 2009). For example, AvrPto binds to the Pto/Prf resistance protein complex that comprises a Pto kinase and the nucleotide-binding leucine rich-repeat protein (NB-LRR) Prf. AvrPto binding to Pto/Prf initiates resistance to *Pst* by stimulating a hypersensitive response (HR) that results in localized cell death (Ntoukakis et al., 2014). Several studies hypothesize that the plant Pto kinase acts as a decoy for AvrPto binding that stimulates a defensive signaling cascade via Prf to induce localized cellular necrosis (van der Hoorn and Kamoun, 2008; Xiang et al., 2008).

AvrPto is targeted to the plasma membrane of plant cells by post-translational modification of its N-terminus (Met-Gly-X-X-Cys) by myristoylation of Gly-2 (following Met-1 cleavage) and palmitoylation of Cys-5 (Shan et al., 2000; de Vries et al., 2006). The addition of 14-carbon and 16-carbon saturated fatty acid moieties to Gly-2 and Cys-5, respectively, confers stable anchoring of the protein to membranes (Resh, 1999) with myristoylation necessary for AvrPto functionality (Shan et al., 2000). Mechanistic studies of AvrPto do not uniformly agree with its deciphered targets and function (Xiang et al., 2010). There is a general consensus that AvrPto binds to the kinase domains of the PRRs FLAGELLIN SENSING 2 (FLS2) and EF-TU RECEPTOR (EFR) to block signaling after perception of the PAMPs flagellin and EF-Tu, respectively (Xiang et al., 2008). Both FLS2 and EFR interact with, and require, the co-receptor BRI1-ASSOCIATED KINASE 1 (BAK1) (Zipfel et al., 2006; Chinchilla et al., 2007; Heese et al., 2007) to form a complex which is required for PAMP triggered immunity (PTI). Extension of this using yeast two hybrid (Y2H) screening approaches in *Arabidopsis thaliana* produced a single common protein from two independent studies (cysteine-rich receptor-like protein kinase 45, At4g11890) (Speth et al., 2009; Mukhtar et al., 2011) and a large number of hits that are potentially false positives due to the nuclear localization of the proteins identified.

The plasma membrane is perhaps the most important signaling interface for plant cells. Plants as sessile organisms must transfer endogenous and environmental signals across this barrier to induce a response. Membrane-imbedded proteins whilst extremely important have historically been difficult to study due to low abundance and the poor solubility associated with hydrophobic transmembrane regions. Although in some cases it has been possible to purify plant plasma membrane proteins and their interactors directly and identify them using mass spectrometry (Gutierrez et al., 2010), the most successful approach has been to use forward genetic screens for specific phenotypes, though this is often not possible.

**FIGURE 1:**
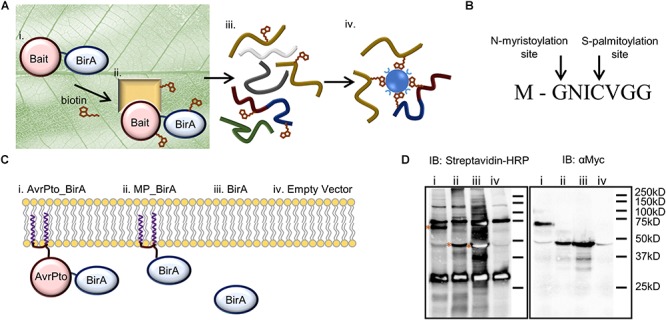
BioID as a technique to identify near neighbor proteins *in vivo* and its adaptation for use in plants to identify AvrPto proximal proteins. **(A)** Identifying protein interactions using BioID includes. (i) Transient expression of the bait protein fused to the biotin ligase within the leaf. (ii) The bait protein interacts with its native binding partners in the plant. Biotin is infiltrated into the leaf where it is activated by the biotin ligase, and then covalently binds to the prey protein. (iii) The leaf tissue is lysed, and the proteins are extracted under denaturing conditions. (iv) Biotinylated proteins are directly purified using streptavidin beads and analyzed by mass spectrometry. **(B)** The 8 amino acid sequence of the N-terminal AvrPto myristoylation and palmitoylation motif. The arrows indicate the sites of co-translational modification. **(C)** Constructs utilized to differentiate interactions (i) that occur with AvrPto_BirA (ii) versus MP_BirA at the plasma membrane (iii) and BirA in the cytoplasm. (iv) The EV control defines natively biotinylated proteins and non-specific interactors. **(D)** Immunoblots (IB) of BioID constructs used to assess the technique. The numbers coincide with the constructs present in **(C)**. The streptavidin-HRP IB detected biotinylated proteins. All BirA constructs were auto-biotinylating as shown by the bands on the streptavidin blot depicted with asterices. The Myc IB reveals protein expression levels for the different constructs.

A more promising approach for detecting protein interactions *in vivo* has recently been developed using biotin ligase (BirA) fusions (Roux et al., 2012). The technique, called BioID, expresses a protein target (the ‘bait’) fused in frame with a mutated form of BirA from *Escherichia coli*. Using biotin as a substrate, BirA generates a reactive biotinoyl-5′-AMP intermediate which is released and covalently bonds to the nearest primary amine - usually a lysine residue or the N-terminus of a neighboring protein labeling these proximal proteins with biotin (Figure 1A). The lifetime/stability of the biotin analog is very limited such that it only interacts with ‘prey’ proteins in very close proximity to BirA, typically within ≤10 nm radius of the BirA-bait fusion protein (Kim et al., 2014). BioID has proven particularly versatile at monitoring prey proteins that interact with membrane bound proteins *in vivo* (Couzens et al., 2013).

The BioID system has previously been utilized in rice protoplasts and most recently in stably transformed Arabidopsis plants (Lin et al., 2017; Khan et al., 2018). Here we have developed the BioID proximity protein labeling approach to work *in planta* and identify plant immune proteins targeted by AvrPto. The system utilizes transient *Agrobacterium tumefaciens* based transformation of *N. benthamiana* to express the BirA fusion proteins in the leaf and requires the exogenous application of biotin for efficient labeling. By expressing a range of BirA fusion proteins within *N. benthamiana* we identify five plant proteins targeted by AvrPto. One protein, NbRIN4, has previously been found to interact with AvrPto and is a central immune regulatory component whilst targeted silencing of the other proteins by viral induced gene silencing (VIGS) reduced the susceptibility of *N. benthamiana* to infection by the bacterial pathogen *P. syringae tabaci*, suggesting roles for the BioID-selected proteins in pathogen defense. Thus, BioID provides a useful addition to methods for studying plasma-membrane associated proteins with potential roles in pathogen defense.

## Results

To set up the BioID system in plants we modified the binary plant expression vector pT70 (Balmuth and Rathjen, 2007) to express bait proteins fused to BirA with a c-Myc tag. The c-Myc tag allowed for western blotting-based assessment of protein expression levels. In order to probe the interactions of AvrPto we designed four constructs which could differentiate between indiscriminate protein interactions at the plasma membrane versus target specific interactions at this interface (Figure 1C). Firstly, AvrPto was fused to the N-terminus of BirA to produce AvrPto_BirA (i). Secondly the 8 amino acid AvrPto myristoylation/palmitoylation (MP) motif was fused to the N-terminus of the BirA protein to produce MP_BirA (ii), targeting the protein to the plasma membrane (Figure 1B). Thirdly BirA was expressed by itself to differentiate interactions in the cytoplasm (iii) and finally an empty vector (EV) control was utilized to differentiate natively biotinylated proteins and non-specific interactions (iv). The four constructs were transiently expressed in *N. benthamiana* leaf tissue using *A. tumefaciens* mediated transformation. Leaf tissue contains a number of natively biotinylated proteins as detected by western blotting using streptavidin-HRP (Figure 1D). Expression of BirA alone in a plant cell results in the labeling of a large number of proteins within the cytoplasm, but significantly less labeling was observed with the BirA fusion constructs.

Retention of AvrPto function when fused to the biotin ligase was confirmed through the use of a hypersensitive response assay. Expression of AvrPto-BirA did not seem to change the function of the protein, as it was still recognized by Pto in *N. benthamiana* producing a severe HR response and no such response was observed in the absence of Pto (Supplementary Figure S1) (Scofield et al., 1996).

**FIGURE 2:**
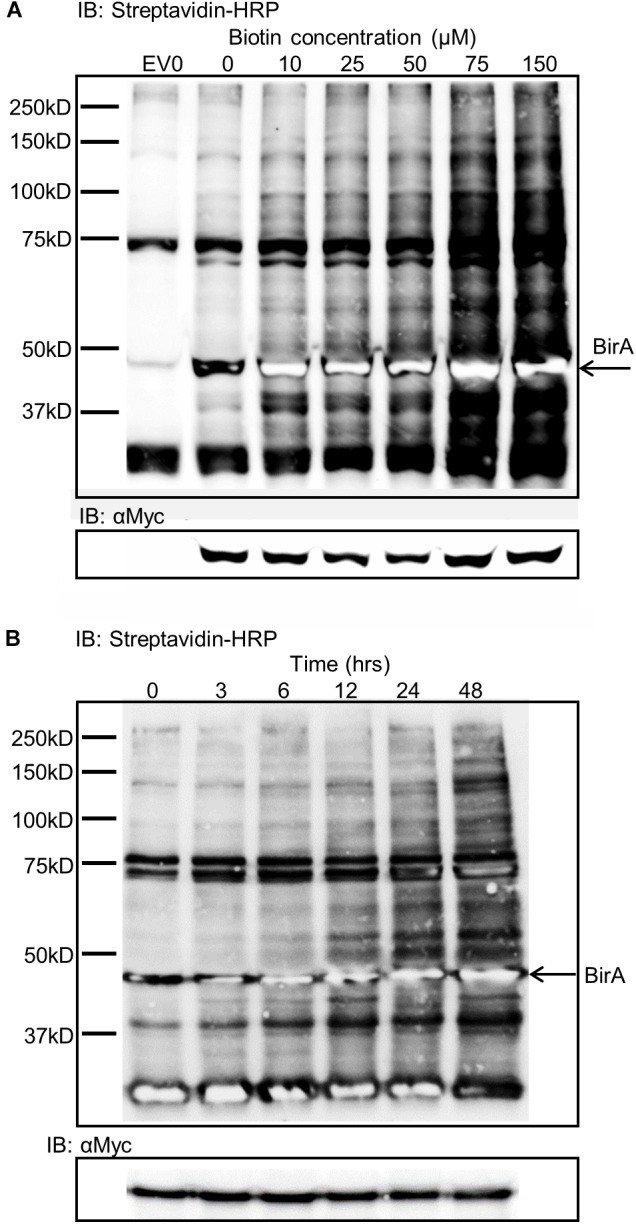
Biotin concentration effects on biotin labeling and time dependence of labeling. **(A)** Streptavidin-HRP IB depicting the level of biotinylation dependent on the concentration of biotin infiltrated into leaf tissue expressing BirA. EV0 represents empty vector with no biotin infiltrated and reveals natively biotinylated proteins. Lanes labeled 0–150 represent leaf tissues expressing BirA with increasing concentrations of biotin infiltrate into the leaf. The Myc immunoblot shows BirA expression levels in the tissues. **(B)** Streptavidin-HRP immunoblot depicting the level of biotinylation of plant proteins by BirA over time. BirA was transiently expressed for 3 days but the time from biotin infiltration until leaf harvest was varied. The Myc immunoblot shows BirA expression levels in the tissues.

Expression of BirA in leaf tissue produced very little labeling without the addition of exogenous biotin substrate (Figure 2A). To overcome the substrate limitation, solutions with varying biotin concentrations from zero to 150 μM were infiltrated directly into the leaf tissue 24 h prior to sampling. The infiltrated biotin was then taken up by the cells to enable BirA-mediated biotinylation. Plants contain a number of natively biotinylated proteins which are observed in all samples including the empty vector sample where BirA was not expressed. The optimum concentration of biotin to infiltrate into the leaf was found to be 75 μM which produced significant labeling of a large range of proteins within the cytoplasm of the plant leaf cells (Figure 2A). Using a time course for biotin infiltration from 3 to 48 h before harvest of the leaf tissue, we found that 24 h was the optimum time point to produce significant biotinylation, though longer time periods did produce more labeling (Figure 2B). For all subsequent experiments *Agrobacterium* was first infiltrated into leaf tissue, then 48 h later a 75 μM biotin solution was similarly infiltrated into the same tissue and the leaves were harvested 24 h after biotin infiltration.

### Proteins Discovered Using BioID

**FIGURE 3:**
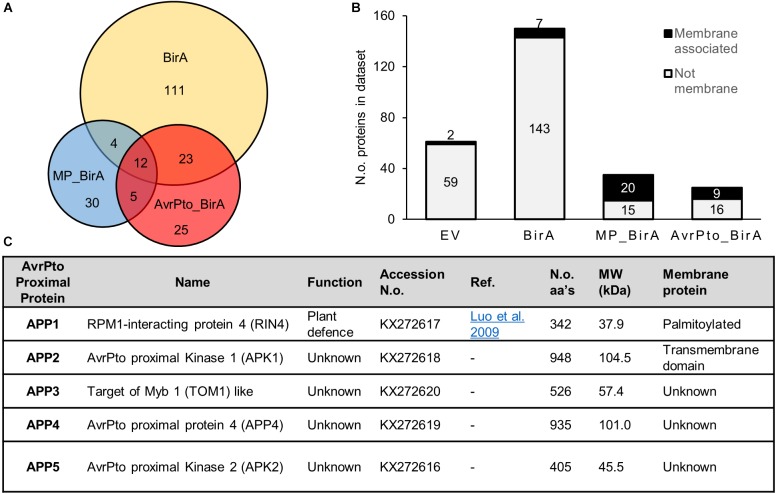
Characteristics of proteins identified using BioID. **(A)** A Venn diagram showing the relative numbers of proteins detected for each construct utilized in the BioID screen. The overlap displays those proteins which were found in multiple constructs. These numbers reflect the total number of identified proteins once the empty vector proteins were removed from the list. **(B)** A graph depicting the number of membrane proteins identified in each dataset. MP_BirA represents those proteins unique to MP_BirA and AvrPto_BirA. The AvrPto BirA column depicts proteins that were unique to the AvrPto_BirA dataset from the three independent experiments. **(C)** Table of the AvrPto proximal proteins identified in this study and their characteristics.

Proteins identified by mass spectrometry (MS) from the BioID experiments were collated into a database containing a total of 271 proteins. The 271 proteins is potentially an exaggerated list as it contains protein isoforms that could not be differentiated by the peptides discovered by MS (Supplementary Table S1). Sixty-one proteins were identified within the EV negative control samples, which includes natively biotinylated proteins, such as acetyl-CoA carboxylase, and proteins non-specifically bound to the streptavidin beads (Supplementary Table S1). The BirA-only control produced a further 150 proteins and all EV and BirA-only hits were then excluded from further analysis (Figure 3A).

Sixty proteins were identified within the AvrPto_BirA and MP_BirA samples with very little overlap observed between the two (Figure 3A). Transmembrane domain prediction was completed using Phobius (Kall et al., 2007) and following manual curation to include known membrane associated proteins, it was found that the vast majority of the predicted membrane proteins were in the MP_BirA and AvrPto_BirA samples (Figure 3B). The bulk of the proteins detected in the BirA-only control samples were predicted to be cytoplasmic as expected which suggests that the system works well for differentiating between membrane and cytosol localizations. Of the 30 proteins within the BirA_AvrPto sample subset, only five proteins appeared in two or more experiments. These five proteins were deemed to be AvrPto proximal proteins (APP) and chosen for further study. Accession numbers and details are given in Figure 3C.

### AvrPto Proximal Proteins

Of the five APP discovered using BioID, APP1 is a known essential regulator of plant defense and plays a central role in resistance upon infection by pathogens (Figure 3C). This protein, RPM1 interacting protein 4 (RIN4), is a negative regulator of plant defense responses and has previously been shown to be a target of AvrPto and several other effector proteins from *Pseudomonas* (Luo et al., 2009). The four remaining APPs have not previously been shown to be targets of effector proteins. APP2 is a leucine rich repeat receptor like protein kinase, which we designate AvrPto-Proximal Kinase 1 (APK1). Interestingly APK1 has an intracellular protein kinase domain that is highly related to Pto, a known target of AvrPto. Alignment of the amino acid sequences highlights the high level of conservation (Supplementary Figure S2). APP3 is annotated as TOM1-like (Target of Myb protein 1) and has 54% amino acid homology with Arabidopsis TOM1-like protein 3 (At1g21380). TOM1 contains both an N-terminal VHS domain and a plant like GAT-GGA domain (Supplementary Figure S3). TOM1 like proteins are suggested to function in the passage of endocytosed ubiquitinated plasma membrane cargo acting as gatekeepers for degradative protein sorting to the vacuole (Korbei et al., 2013; Valencia et al., 2016). APP4 has no assigned function but is predicted to contain a zinc finger domain at the N-terminus and contains some homology with the pentatricopeptide repeat superfamily. This gene is highly conserved in Solanaceae but only the N and C-terminal regions retain any conservation in other plant families. The closest Arabidopsis homolog to APP4 is At4g14200 which is a pentatricopeptide repeat protein, but these proteins share only 18% amino acid identity. APP5 is a serine threonine protein kinase which we name AvrPto Proximal Kinase 2 (APK2) and is most closely related to the Arabidopsis protein At3g22750 with 72% amino acid identity. At3g22750 is a MAPKKK of the Raf subfamily with the common name Raf39 (Jonak et al., 2002). APK2 contains the GTxx(W/Y)MAPE motif within the kinase domain which also places it in the Raf subfamily.

### Validation of AvrPto Proximal Protein Biotinylation

**FIGURE 4:**
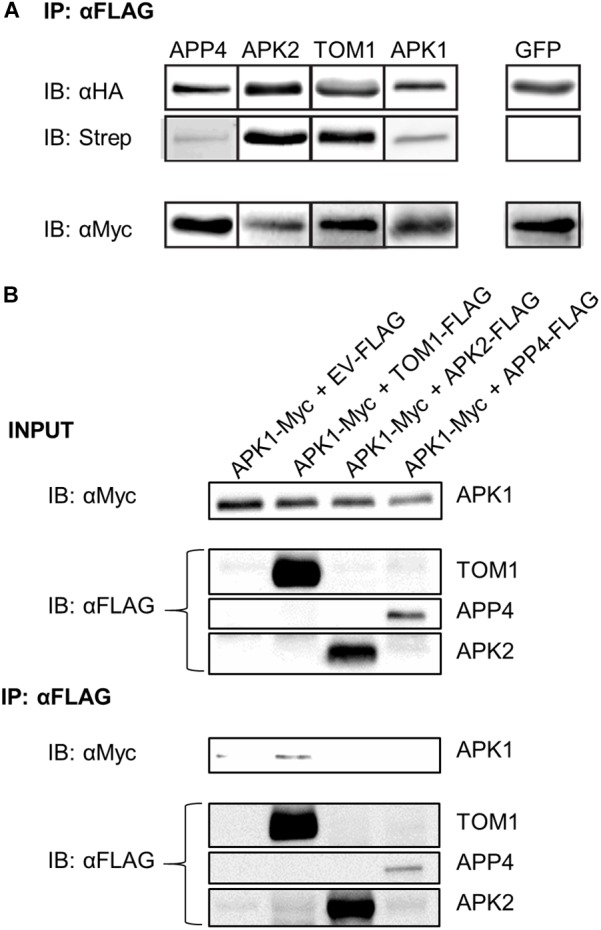
Biotinylation assay of APP’s and detection of protein–protein interactions. **(A)** Biotinylation assay of AvrPto proximal proteins. APPs 2-5 were expressed with HA_FLAG tags along with AvrPto_BirA-Myc. The APPs were immunoprecipitated using αFLAG beads. The immunoprecipitated proteins were immunoblotted using αHA antibodies. To determine if the immunoprecipitated proteins were biotinylated the blots were probed with streptavidin-HRP. The expression levels of AvrPto_BirA were determined by probing with αMyc antibodies. All four expressed APPs were found to be biotinylated whereas the control GFP protein was not. This experiment was repeated 3 times and the typical result is shown. The figure is cut down to simplify understanding. **(B)** Co-immunoprecipitation of APPs reveals interaction between APK1 and TOM1. The AvrPto proximal proteins were transiently expressed with HA-FLAG tags in the presence of APK1-Myc in *N. benthamiana*. The FLAG tagged proteins were immunoprecipitated with αFLAG beads and the presence of APK1 was probed by immunoblotting with αMyc.

To further study APPs 2-5 (RIN4 was excluded because it is well characterized) (Mackey et al., 2002; Liu et al., 2009, 2011; Luo et al., 2009) they were amplified and cloned from *N. benthamiana* cDNA into binary expression vectors encoding C-terminal affinity tags. We next devised a protocol to detect AvrPto-dependent biotinylation independent of mass spectrometry. As such the four novel AvrPto proximal proteins were expressed transiently in *N. benthamiana* in the presence of AvrPto_BirA. The proteins were then immunoprecipitated via the FLAG tag and probed to detect biotinylation using western blotting (Figure 4A). This assay confirmed that all 4 APPs were biotinylated in the presence of AvrPto_BirA, but the GFP control was not.

### Co-immunoprecipitation of AvrPto Proximal Proteins

We used coimmunoprecipitation (CoIP) assays to test potential protein–protein interactions between AvrPto and each APP. Using pairwise assays, we epitope-tagged each APP and co-expressed them with AvrPto by transient transformation in *N. benthamiana*. CoIP’s were performed in both orientations. We detected no interactions in these assays (data not shown). Systematic testing of potential interactions between all APPs did, however, reveal an interaction between two of these proteins (Figure 4B). APK1-Myc was co-expressed in *N. benthamiana* leaves with either an EV control or with TOM1-FLAG, APK2-FLAG, or APP4-FLAG. The accumulation of these proteins was assessed using western blotting as shown in Figure 4B INPUT. Immunoprecipitation with anti-FLAG beads followed by western blot detection of Myc-tagged proteins revealed that APK1-Myc was coimmunoprecipitated with TOM1-FLAG (Figure 4B IB: αFLAG). This interaction could also be repeated in reverse CoIP’s where APK1-Myc was used as the bait to pull down TOM1-FLAG (data not shown). Therefore, this seems to be a robust interaction.

### *APP* Gene Regulation in Response to *Pst* DC3000 and Transient *AvrPto* Expression

**FIGURE 5:**
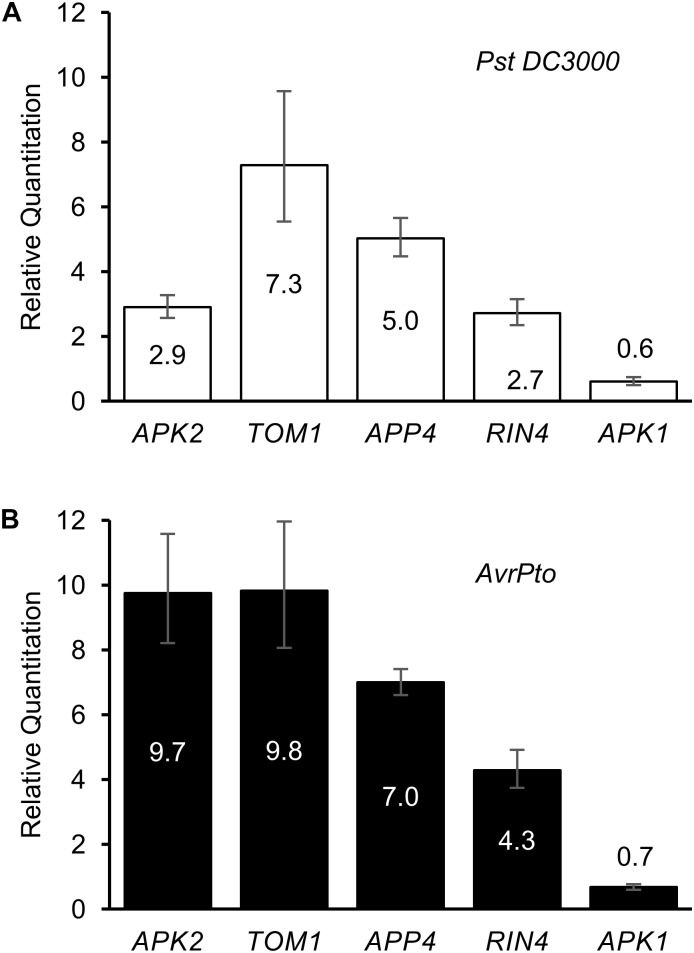
Regulation of AvrPto proximal proteins in response to elicitors. Relative quantitation of gene expression levels in response to **(A)** *Pst* DC3000 infiltration and to **(B)** AvrPto expressed by *A. tumefaciens*. Controls used were water for the bacterial infiltration and *A. tumefaciens* expressing an empty vector for the AvrPto samples.

Regulation of the five *APP* genes was determined in response to *Pst* DC3000 infection, and separately to transient expression of the *Pst* effector protein AvrPto (Figure 5A). Twenty four hours after infection with *Pst* DC3000 (which expresses a genomic copy of *AvrPto*), and before the onset of cell death due to the recognition of *HopQ1* (Wei et al., 2007), the genes encoding *RIN4, TOM1, APP4,* and *APK2* were found to be significantly up-regulated, whereas *APK1* expression levels dropped slightly over this time period. *RIN4* has previously been reported to be upregulated in response to *P. syringae* infection (Luo et al., 2009). When AvrPto was expressed transiently in *N. benthamiana* leaves using *A. tumefaciens* mediated transformation, the *APP* genes were even more highly up-regulated when compared to the EV control (Figure 5B). Thus, APP genes are responsive to both pathogen infection and transient transformation with the *AvrPto* gene.

### The Involvement of AvrPto Proximal Proteins in Immunity

To test the involvement of the APPs in immunity these genes were selectively silenced in *N. benthamiana* plants using virus-induced gene silencing (Senthil-Kumar and Mysore, 2014). Silencing was successful for all *APPs* (Supplementary Figure S4) except for the kinase gene *APK2* where the construct that we used failed to knock down gene expression. It is worth noting that the silencing of *APP4* resulted in a severely altered phenotype with the plants appearing stunted and producing significantly less above-ground biomass compared to the *GFP*-silenced controls (Supplementary Figure S5).

**FIGURE 6:**
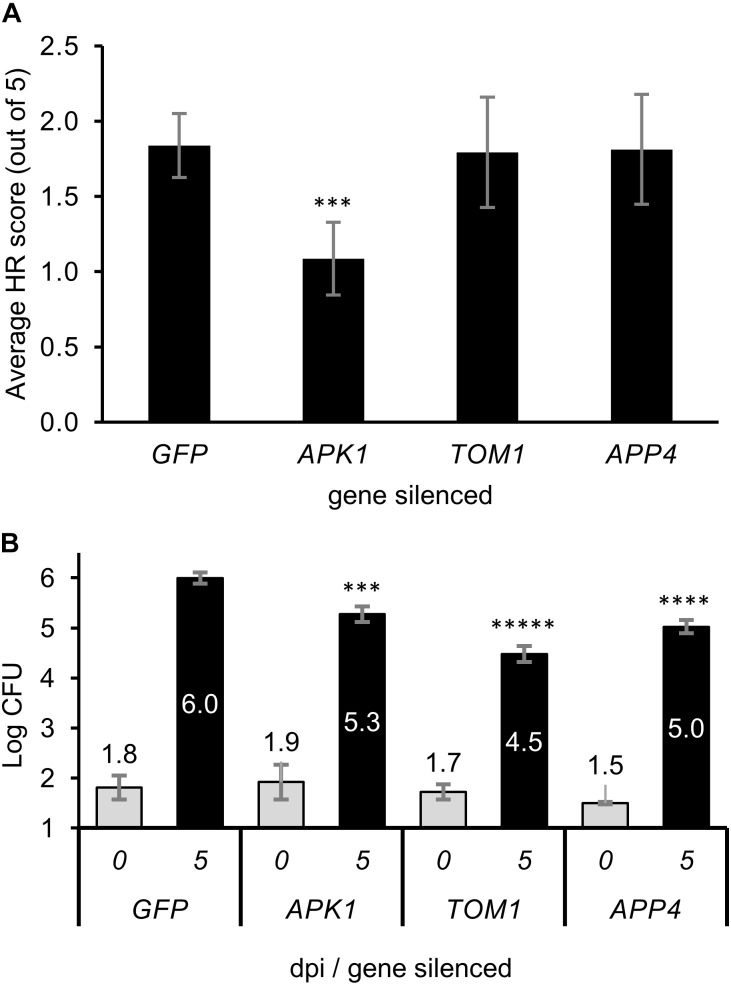
The effect of virus induced gene silencing of the APPs on plant immunity. **(A)** The level of hypersensitive response observed when the Pto/Prf genes were co-expressed in *N. benthamiana* plants which were silenced for individual *APP* genes. *A. tumefaciens* was infiltrated into the leaves to transiently express Pto/Prf and the results shown are 5 dpi. The hypersensitive response was scored visually from 0 to 5 with a minimum of eight replicates from multiple biological experiments. **(B)** Pathogen growth assay on plants silenced for APPs. *N. benthamiana* plants were silenced for individual *APP* genes using VIGS then infected with *Pseudomonas syringae tabaci*. Leaf tissue for bacterial assays was harvested 5 dpi and assayed. ^∗∗∗^*p* < 0.001, ^∗∗∗∗^*p* < 1 × 10^-5^, ^∗∗∗∗∗^*p* < 1 × 10^-7^.

Transient expression of the tomato *Pto/Prf* genes in *N. benthamiana* normally results in a weak autoactive HR response (Mucyn et al., 2006). As such. We expressed the *Pto/Prf* genes in leaf tissue silenced for each *APP*, and HR was scored by visual examination of the abaxial side of the leaf tissue (Figure 6A and Supplementary Figure S6). The silencing of *APK1* was found to significantly diminish the *Pto/Prf*-induced HR, whilst silencing of *TOM1* or *APP4* did not affect this response. Overexpression of the four functionally uncharacterized APPs using Agrobacterium-mediated transient gene expression in *N. benthamiana* by themselves or in combination with each other did not induce HR, or effect the AvrPto-dependent or *Pto/Prf* induced HR.

The significance of *APPs* in immunity was tested by silencing the genes individually and infecting silenced plants with *P. syringae* pv. *tabaci* (*Pstab*). The growth of *Pstab* on plants silenced for the *APP* genes or the control gene *GFP* (which is not present in the plant) resulted in a statistically significant decrease in growth of the pathogen for *APK1, TOM1,* and *APP4* compared to the control plants (Figure 6B). Plants silenced for *TOM1* showed the most significant decrease in growth of the bacteria. The decrease in pathogen growth is similar to that observed by Luo et al. (2009) for RIN4 which is a negative regulator of plant defense and whose knockdown resulted in decreased susceptibility to pathogen growth.

## Discussion

We describe here adaptation of the BioID system for rapid detection of proteins proximal to a protein bait in live plant tissue. Utilizing transient *Agrobacterium-*based gene expression removes the need to construct stably transformed plants. This makes the technique relatively quick and easy to use. The introduction of biotin into the leaf by infiltration also provides a simple way to supply the substrate. Once the transient expression of proteins was optimized and favorable biotin concentrations were determined, the key innovation in adapting BioID for use in live plants was the development of a rapid methodology to remove excess biotin from leaf extracts using a disposable size exclusion column (see section “Materials and Methods” section).

The use of the BioID system for labeling proximal proteins within plant cells was found to be both time and substrate concentration dependent revealing that the BirA enzyme is rate limited by access to the substrate within plant tissues. Increases in biotinylation through increased substrate concentration were observed up to 500 μM, but at the highest biotin concentrations transient expression of the bait protein was inhibited.

We observed that BirA expressed alone labeled more proteins than did the fusion proteins, as observed by western blot (Figure 1D) and by the number of proteins discovered using mass spectrometry (Figure 3A). This can be partly explained by the higher levels of expression observed for BirA. It is also likely due to the fact that the fusion proteins are directed to a specific region within the cell, thus reducing the subset of proteins they can interact with. This is supported by the reduction in the number of proteins labeled when BirA was directed to the plasma membrane (MP_BirA) where it is presumably restricted in the number of proteins available for interaction. Additionally, this restriction was further exaggerated when AvrPto was fused to BirA, with even fewer hits identified (Figures 1D, 3A,B). The EV and BirA constructs produced a significant list of proteins which were excluded from all further analysis. This list of background contaminants will be of use for future experiments to avoid false positives (Supplementary Table S1).

Of the total dataset of biotinylated proteins, we found that the vast majority of the predicted membrane proteins were in the MP_BirA and AvrPto_BirA samples (Figure 3B). This corroborates the evidence that AvrPto is found at the plasma membrane. The lack of overlap observed for the hits from the AvrPto_BirA and MP_BirA constructs (Figure 3A) is consistent with AvrPto_BirA specifically labeling proteins in close proximity to AvrPto and not all proteins at the plasma membrane, confirming the high spatial resolution of the BioID technique (Van Itallie et al., 2013).

Of the five APPs discovered using BioID it was encouraging that RIN4 (APP1) has previously be shown to interact with AvrPto using both Y2H and co-immunoprecipitation techniques (Luo et al., 2009). Additionally, it is worth noting that this protein is also found at the plasma membrane, anchored in place by a C-terminal palmitoylation group (Kim et al., 2005). RIN4 is degraded in the presence of AvrPto and this degradation in *Solanaceous* plants is dependent on the Pto/Prf resistance protein complex. Additionally, the degradation of RIN4 is reliant on the presence of conserved AvrRpt2 cleavage sites at the N and C-terminus (Ntoukakis et al., 2014). NbRIN4 contains the conserved AvrRpt cleavage sites at the N and C-terminus and has 79% amino acid identity to *Solanum lycopersicum* RIN4 which has been extensively studied (TC174419) (Luo et al., 2009).

APK1 (APP2) is a member of the leucine rich repeat (LRR) VIII class of LRR-RKs, which have not previously been implicated in immunity. (Monaghan and Zipfel, 2012). APK1 from *N. benthamiana* is most closely related to the Arabidopsis gene At5g49760 and contains 55% amino acid identity. APK1 is predicted to encode a transit peptide followed by an extracellular region containing 14 leucine rich repeats, a transmembrane domain, and an intracellular protein tyrosine kinase domain. The kinase domain of APK1 has high homology to Pto which is a known target of AvrPto. This suggests that APK1 may also act as a bait for AvrPto (Supplementary Figure S2), although this could not be confirmed by coimmunoprecipitation.

TOM1 (APP3) contains both a VHS domain and a plant like GAT-GGA domain and has 23% homology with the closest human homolog, TOM1-like protein 2 isoform 3 (NP_001076437.1). For human TOM1, the VHS domain is associated with the plasma membrane (Misra et al., 2000) and contains a positively charged phosphatidylinositol 5-phosphate (PtdIns5P) binding motif. The PtdIns5P is responsible for recruiting TOM1 onto signaling endosomes and regulates endosomal maturation (Boal et al., 2015). We found that the positively charged PtdIns5P binding motif is conserved in NbTOM1 (Supplementary Figure S3), suggesting that TOM1 in plants could perform a similar role in PtdIns5P signaling pathways. The pathways so far found to be regulated by PtdIns5P and its binding proteins in mammalian cells include responses to pathogen invasion, stress responses, apoptosis and autophagosome biogenesis (Shisheva, 2013; Vicinanza et al., 2015). NbTOM1 also has a plant like GAT-GGA domain which in mammalian TOM1 is critical for trafficking soluble proteins from the *trans*-Golgi network to endosomes/lysosomes through interactions with clathrin, GTPases, and TGN-sorting receptors (Shiba et al., 2003). In plants it has recently been shown that clathrin dependent internalization of ligand-activated receptor kinases into the endosomal pathway is required to activate immunity (Mbengue et al., 2016).

The most closely related Arabidopsis gene to *APP4* is At4g14200. This protein is documented as being a pentatricopeptide repeat protein (PPR) which in Arabidopsis makes it a member of one of the largest gene families. PPRs have been demonstrated to have a role in plant growth and development. The Arabidopsis PPR protein called PENTATRICOPEPTIDE REPEAT PROTEIN FOR GERMINATION ON NaCl (PGN) has been shown to function in plant defense against a necrotrophic fungus and is involved in abiotic stress tolerance (Laluk et al., 2011). The knockdown of *APP4* produced a severely stunted phenotype and inhibited growth of *Pstab* on *N. benthamiana*, and the precedent for the involvement of related genes in immunity and stress regulation point to it having a related function.

APK2 (APP5) which is a MAPKKK is most closely related to the Arabidopsis gene At3g22750. At3g22750 was identified in a phosphoproteomic screen of plasma membrane proteins present after treatment with the fungal elicitor xylanase (along with RIN4 in the same study) (Benschop et al., 2007). This protein will be of interest in follow up studies to determine its function *in planta*.

The upregulation of the *APP* genes in response to pathogenic bacteria, and even more so to AvrPto (Figure 5), suggests that they are specifically upregulated in response to the effector. This might be expected for genes encoding immune proteins that are targeted by AvrPto. AvrPto did not interact directly interact with APPs using CoIP assays. This may be because BioID is able to detect weak or transient interactions which would normally be missed by CoIP, or because it detects proximity rather than direct interactions *per se*. Encouragingly, we found that two APPs, APK1, and TOM1, interacted with each other (Figure 4B). This is likely the result of the AvrPto_BirA protein labeling multiple members of a protein complex at the plasma membrane. The fact that APK1 and TOM1 form a complex at the plasma membrane implies that they work cooperatively in the immune response. AvrPto’s tendency to target the kinase domains of receptor like kinases (Xiang et al., 2008) would point to APK1 as the binding target.

The significant decrease in bacterial growth on plants silenced for either *TOM1* or *APK1* suggests that this protein complex acts to negatively regulate the immune response to *Pstab* in *N. benthamiana*. Silencing of *TOM1* was found to have a greater effect on inhibition of bacterial growth than *APK1*, and as such may play a more important role in plant immunity. Something worth following in later work would be to determine if TOM1 has a role in recruitment of the activated receptor-like kinase to the endosomal pathway to facilitate signaling, thus linking the receptor to the clathrin dependent internalization pathway (Mbengue et al., 2016).

Silencing *APP4* produced significantly less bacterial growth, which may be evidence for immune related function but could also be biased by the stunted phenotype. The decrease in bacterial growth when *APP4* was silenced again suggests negative regulation of the immune response in *N. benthamiana*.

Comparison of this study with previous uses of the BioID technology for assessing protein–protein interactions reveals some significant differences in approach. The first use of BioID published in plants (Lin et al., 2017) utilized protoplast transformation which simplifies the process of getting the biotin substrate into the cells and also of removing extraneous biotin before affinity capture of biotinylated proteins. The downside of this technique is the difficulty of making protoplasts and the fact that the plant cells are not in their native state which may change protein expression and interactions especially for immune related proteins which may sense cell wall status. The second published study of BioID in plants (Khan et al., 2018) utilized stably transformed Arabidopsis plants and infiltrated biotin into the leaves to achieve biotinylation. This process involves making stably transformed plants which enables biotin labeling to occur in the whole leaf. The major drawback of this technique is the laborious and time-consuming effort required to make stably transformed plants. Khan et al. (2018) also use this system to determine the interactions of an effector protein at the plasma membrane but fail to include the critical control where BirA is targeted to the plasma membrane which would potentially eliminate more false positives. Khan et al. (2018) utilize centrifugal filtration to buffer exchange the sample and remove biotin. We found this to take too long and didn’t remove all biotin and so utilized single use size exclusion columns to remove biotin from the sample before affinity capture of biotinylated proteins. Khan et al. (2018) infiltrated 2 mm biotin into the leaf to promote biotinylation by BirA; we found that at such high concentrations Agrobacterium based transient expression of proteins was diminished and so utilized lower concentrations of biotin.

## Conclusion

We have developed a novel strategy to track proteins proximal to a bait protein within living plant tissue. It is particularly amenable for users to determine the interactions between pathogenic effector proteins and plant host molecules. We have used this technique through the AvrPto effector protein to delineate several new proteins which are potentially involved in the plant immune network. Two of the most interesting proteins found in this study were APK1 and TOM1, which form a stable complex with each other at the plasma membrane. Silencing either gene resulted in reduced growth of pathogenic bacteria, suggesting that they act to negatively regulate the plant immune response.

BioID is a powerful new technique with high potential for determining novel plant protein interactions. The ability to tag proximal prey proteins with biotin then directly isolate them at a later time point under denaturing conditions is a major advantage compared to traditional co-immunoprecipitation techniques, which rely on maintaining the interaction during purification. The technique appears to be particularly good for defining membrane protein interactions at the plasma membrane interface. Though the technique has been used here to delineate the plant immune proteins targeted by bacterial pathogens it will be useful for finding a whole range of other protein–protein interactions in plants.

## Materials and Methods

### Transient Expression of Fusion Proteins for BioID

Growth and transient expression conditions for *N. benthamiana* were as described (Wu et al., 2004) using the *A. tumefaciens* strain GV3101 pMp90. *N. benthamiana* leaves were infiltrated with *A. tumefaciens* cultures (OD_600__nm_ = 0.5–1) containing the pT70 BirA_c-Myc vector constructs. The pT70 BirA vector utilized a codon optimized BirA gene, with the required R118G substitution to promote release of the reactive biotin and encodes the BirA gene as a C-terminal fusion protein with a 5 × c-Myc epitope tag. Twenty-four to forty-eight hours after infiltration with *A. tumefaciens,* a 75 μM solution of biotin was infiltrated into the whole leaf. The leaf tissue was harvested 24 h later and frozen at -80°C until needed.

### Protein Extraction for BioID

Leaf material [300 mg) was ground in liquid nitrogen and proteins were then extracted in 1.8 ml of chilled extraction buffer (150 mM Tris-HCl pH 7.5, 500 mM NaCl, 0.4% SDS, 2% Triton X-100, 10 mM EDTA, 15 mM DTT, 2% insoluble polyvinylpolypyrrolidone (PVPP), 1% plant proteinase inhibitor cocktail] by vortexing briefly. Precipitates was removed by centrifugation at 15,000 *g* for 5 min at 4°C, supernatants were then transferred to a new tube and centrifuged for 15,000 *g* for 20 min at 4°C. Supernatants were then subjected to filtration through a 0.2 μM syringe filter to remove any particulate material.

### Removal of Free Biotin

The infiltration of the leaves with biotin produces a large excess of biotin in the extracted protein samples preventing efficient pull down of the biotinylated proteins. To remove unbound biotin from the samples the supernatants were run onto a PD10 size exclusion column (GE Healthcare Life Sciences) which was pre-equilibrated with elution buffer; 150 mM Tris-HCl pH 7.5, 500 mM NaCl, 0.4% SDS, 1% Triton X-100, 10 mM EDTA. The proteins were eluted in 3.2 ml of extraction buffer and the flow through was collected. The addition of a step to remove excess free biotin significantly increased the efficiency of the streptavidin based affinity purification.

### Immunoprecipitation of Biotinylated Proteins

Streptavidin coated Dynabeads (300 μL/sample, MyOne streptavidin C1, Thermo Fisher) were added to the PD10 column eluate and the samples were incubated spinning slowly on a wheel at 4°C for 3 h. The beads were separated from the supernatant using a Dyna-Mag magnet (Invitrogen) and washed extensively as described (Roux et al., 2012). Washed beads were frozen at -80°C before analysis by MS. Ten percent of the beads were taken and run on SDS-PAGE gels for western blot analysis. Beads were resuspended in SDS PAGE sample buffer containing 1 mM biotin and 10 mM DTT and heated at 95°C for 10 min before separation by SDS-PAGE.

### Identification of Proteins by Mass Spectrometry

The Dynabeads were re-suspended in 100 mM triethylammonium bicarbonate buffer (TEAB, pH 8.5) and biotinylated proteins were reduced with 5 mM Tris (2-carboxy ethyl) phosphine hydrochloride for 30 min at 25°C, followed by alkylation with 10 mM iodoacetamide for 45 min at 25°C in the dark. The beads were then washed three times with 100 mM TEAB. The proteins on the beads were digested in 50 mM TEAB, 5% acetonitrile using a modified trypsin (Roche, sequencing grade) to protein ratio of 1:50 overnight at 37°C. The peptides were acidified with formic acid to a 5% final concentration.

For LC-MS/MS analysis tryptic peptides were analyzed on a nanoACQUITY UPLC system (Waters Corporation, Milford, MA, United States) coupled either to an LTQ-Velos Pro or an Elite Orbitrap mass spectrometer (Thermo Fisher Scientific, Bremen, Germany). Mobile phases for chromatographic peptide separation were as follows: Eluent A was 0.1% formic acid and eluent B was acetonitrile with 0.1% formic acid. Acidified samples were run on a reversed-phase trap column (Symmetry C18 5 μm, 180 μm × 20 mm; Waters) and washed for 3 min at 15 μL/min using 2% eluent B. Peptide mixtures were subsequently flushed onto a capillary column (BEH130 C18 1.7 μm, 75 μm × 200 mm; Waters) and separated applying the following gradient at a flow rate of 300 nL/min and column temperature at 35°C: 0 min (2% eluent B) – 1 min (5% B) – 91 min (30% B) – 101 min (45% B) – 108 min (95% B) – 118 min (95% B) – 120 min (2% B) – 140 min (2% B).

Column-separated peptides were run through a Nanospray Flex Ion Source (Thermo Fisher Scientific). Spray voltage was 1.8 kV with no sheath, sweep or auxiliary gasses used. The capillary temperature was set to 285°C and the S-lens to 55%. The mass spectrometer was controlled in positive ion and data-dependent acquisition mode to automatically switch between Orbitrap-full scan MS and ion trap- MS/MS acquisition. Full scan MS spectra (*m/z* 380 – 1700) were acquired in the Orbitrap mass analyzer with a resolving power set to 60,000 and 120,000 (at 400 *m/z*) for Velos Pro and Elite, respectively, after accumulation to a target value of 1 × 10^6^ in the linear ion trap. The top 15 most intense ions with charge states ≥ +2 were sequentially isolated with a target value of 5,000 and fragmented using collision-induced dissociation (CID) in the linear ion trap. Fragmentation conditions were set as follows: 35% normalized collision energy; activation q of 0.25; 10 ms activation time; ion selection threshold 500 counts. Maximum ion injection times were 200 ms for survey full scans and 50 ms for MS/MS scans. Dynamic exclusion was set to 90 s. Lock mass of *m*/*z* 445.12 was applied with an abundance set at 0%.

For database searching; mass spectra were converted into Mascot format and Mascot (Matrix Science, version 2.4) was employed to match MS/MS peak lists to a 6-frame translation of the *N. benthamiana* v5 transcriptome (238,123 entries) (Nakasugi et al., 2014), supplemented with expressed sequences, and a contaminants database ^1^. Mascot searches were performed assuming trypsin digestion with two missed cleavages, a fragment ion mass tolerance of 0.80 Da and a parent ion tolerance of 20 ppm.

Criteria for protein identification; Scaffold (Proteome Software Inc., version 4.3.4) was used to validate MS/MS based peptide and protein identifications. Peptide identifications required greater than 95.0% probability using the Peptide Prophet algorithm (Keller et al., 2002) and protein identifications required a greater than 99.0% probability (Nesvizhskii et al., 2003) to achieve an FDR less than 1.0%. Identified proteins required a minimum of two identified peptides. Proteins that contained similar peptides and could not be differentiated based on MS/MS analysis alone were grouped to satisfy the principles of parsimony.

### Interaction Scoring for BioID

The BioID interaction data set comprises samples from *N. benthamiana* with biological replicates generated on separate days in different plants for each bait protein. Several negative controls were used, including EV and BirA alone. The negative control experiments were performed on a minimum of two independent biological replicates. All identified proteins were identified based on a minimum of two peptides with >95% probability giving high confidence hits only. All proteins identified in EV controls were removed from the list of identified proteins for all samples as they are either non-specifically bound contaminants or natively biotinylated proteins. Interactions found in the effector protein samples were from three independent experiments using separate biological replicates and these interactors were collapsed to a single isoform for further analysis.

### Co-immunoprecipitation Assays

Co-immunoprecipitation assays were performed as described (Saur et al., 2015).

### Biotinylation Assay for AvrPto Proximal Proteins

To validate the four novel AvrPto proximal proteins each gene was cloned from *N. benthamiana* cDNA into binary expression vector encoding a C-terminal 3xHA-FLAG affinity tag. These proteins were then expressed transiently in *N. benthamiana* in the presence of AvrPto_BirA. Immunoprecipitation of the APPs using α-FLAG beads followed by western blotting detection with αHA antibodies was used to ascertain that the proteins immunoprecipitated successfully. The precipitated proteins were then probed to detect biotinylation using Streptavidin-HRP. This assay was performed a minimum of three times and results shown are typical.

### Quantitative Real Time PCR

RNA was extracted from leaf tissue using a Qiagen RNAeasy plant mini kit and cDNA was made from the RNA using superscript III (Invitrogen) as per instructions. cDNA was prepared from three separate plant tissue samples for each treatment and quantitative RT-PCR was completed on each biological sample with three technical replicates for each gene within a sample. All expression data were normali007Aed using the *EF1α* housekeeping gene (Heese et al., 2007).

Quantitative RT-PCR was performed using the following primers:

APP1 FWD AAGTGCGGGATCTGAACATAG
APP1 REV CTTTCCTCCCGAACCTTGTTA
APP2 FWD GGTGGTTATGGCAAGGTTTAC
APP2 REV GGCTATCTTCAGTCTCCTCATC
APP3 FWD TCAAGTCCCAAGCCTTCTTC
APP3 REV TGTTTGTCCAACTACGCTATCA
APP4 FWD GTTTACGAGCCTCCGTCTTT
APP4 REV GATTTGGCTTCACCGAGTAGAT
APP5 FWD GGGCACCTATGGTACTGTTTAT
APP5 REV CAGGGAGGAATTCCACAAGAA

### Accession Numbers

Sequence data from this article can be found in the GenBank data library under the following accession numbers: Prf (Q96485), Pto (Q40234), AvrPto (Q87Y16).

### Pathogen Growth Assay

*Nicotiana benthamiana* plants were silenced for either APP2, APP3, APP4, APP5, or GFP. Leaves of silenced plants were dipped in *P. syringae* pv. *tabaci* cultures (OD_600_ = 0.1), containing 10 mM MgCl2 and 0.002% Silwett L-77 for 30 s. Infected leaves were kept under humid conditions for 5 days before taking leaf disks from a minimum of eight leaves. Each set of leaf disks was ground in 10 mM MgCl_2_ and these solutions were serially diluted at 10-fold dilutions. Diluted cultures were plated onto LB agar containing Rifampicin (100 μg/ml) and grown at 28°C for 48 h. Colonies were counted and the number of colony forming units was determined.

### Cloning *APP* Genes

Total RNA was extracted from *N. benthamiana* leaf tissue using the RNeasy kit (Qiagen) and cDNA was generated using SuperScript III (Thermo Fisher) according to the manufacturer’s instructions. The following primers were used to amplify the genes from *N. benthamiana* cDNA:

APP2_Fwd GTCGACCTCGAGATGAGAATTCAGCTTTG TTTTCTGG
APP2_Rev ACTAGTTCTAGACTTGGGCTCTAACTTTGA AGGTGG
APP3_Fwd CTCGAGGTCGACATGGCTAATAATGCAGCA GCTTGTG
APP3_Rev ACTAGTTCTAGAAAATGATCTATTGGATTG GTTTGAC
APP4_Fwd CTCGAGGTCGACATGGATATCCAAGATCAC AAATTTAC
APP4_Rev ACTAGTTCTAGATGCGTCTTGATGTACTGA AGAGCAG
APP5_Fwd CTCGAGGTCGACATGGATTTGAAAAGTGA GGAGAAGG
APP5_Rev ACTAGTTCTAGATGGACCCCTGGTAGGAGC AAAGCAG

All constructs were cloned into the binary vector pT70 (a pTFS-40 derivative containing the 35S promoter) encoding either 5xMyc or 3xHA-1xFLAG (3xHAF) C-terminal epitope tags and transformed into *A. tumefaciens* GV3101 pMp90 (Balmuth and Rathjen, 2007; Gutierrez et al., 2010). Agroinfiltration for transient protein expression was performed as described (Mucyn et al., 2006).

### Virus Induced Gene Silencing

Sequences corresponding to five of the AvrPto proximal proteins were cloned into the silencing vector pYY13 using ligation independent cloning methods (Dong et al., 2007) after amplifying with the following primers.

APP2-Fwd CGACGACAAGACCCTCTCGTGGAGAACAA CAAGCTTAC
APP2-Rev GAGGAGAAGAGCCCTCTCATATTGACAGT CTGCAACTG
APP3-Fwd CGACGACAAGACCCTCTCTTGAATCAGAT CCTTCTGGAC
APP3-Rev GAGGAGAAGAGCCCTGAGAGAAATCATCT TCTGACTCATC
APP4-Fwd CGACGACAAGACCCTGACGTCAGATAATG CAAGTTATG
APP4-Rev GAGGAGAAGAGCCCTCACCACATGCACAT CTTCCCTTAC

The APP5 primers produced a VIGS construct which did not silence the gene.

APP5-Fwd CGACGACAAGACCCTCATAGACCCAAGGA AGTATGGGAG
APP5-Rev GAGGAGAAGAGCCCTGTTCCACCAGGGAG GAATTCCAC

The genes encoding the APP proteins of interest were silenced in young *N. benthamiana* plants using VIGS as described (Heese et al., 2007).

## Author Contributions

BC and JR designed the study. BC completed the study and wrote the manuscript. TS and JG completed the mass spectrometry. IS provided critical feedback on the manuscript.

## Conflict of Interest Statement

The authors declare that the research was conducted in the absence of any commercial or financial relationships that could be construed as a potential conflict of interest.

## Footnotes

1 maxquant.org

## Supplementary Material

FIGURE S1 Expression of AvrPto_BirA fusion in *N. benthamiana*. Transient expression of AvrPto_BirA in *N. benthamiana* revealed that the protein produces a strong hypersensitive response when expressed with Pto. The photo was taken 5 dpi.

FIGURE S2 Alignment of APK1 and tomato Pto kinase, a known AvrPto interactor. The kinase domains contain 45% amino acid identity and 63% positive identities.

FIGURE S3 Alignment of TOM1 from *N. benthamiana*, *A. thaliana* and *Homo sapiens*. The alignment of the amino acid sequences of TOM1 showing the conservation of the VHS and GAT GGA domains as well as the PtdIns5P binding motif. NbTOM1 (KX272620), AtTOM1 (At1g21380), HsTOM1 (NP_001076437.1).

FIGURE S4 Effectiveness of VIGS based gene silencing of *N. benthamiana* *APP* genes. Q-RT PCR analysis of *APP* gene expression in *N. benthamiana* plants that were silenced for the individual *APP* genes.

FIGURE S5 Above ground biomass of *N. benthamiana* plants silenced for *APP* genes. Plants silenced for the *APP* genes were harvested at 7 weeks of age and the above ground wet biomass was measured. The ^∗^ indicates a *p*-value < 0.05 as compared to untreated wild type plants.

FIGURE S6 Representation of hypersensitive response scoring system. The HR score ranged from 1 which is the beginning of the herpsensitive response showing a discernible collapse of the tissue and a shiny appearance, to 5 which is complete necrosis with the leaf tissue appearing dried and dead.

TABLE S1 Proteins identified by Mass spectrometry. Accession numbers, homology details and aminoacid sequence are provided for each protein identified by MS, along with the number of peptides detected in each sample. The table is colour coded to indicate which sample the peptides were detected in.
