# Supplementary figures and images for: Development of a Rapid in planta BioID System as a Probe for Plasma Membrane-Associated Immunity Proteins
Source: Front Plant Sci. 2018 Dec 18;9:1882. doi: 10.3389/fpls.2018.01882 (PMC6305590; doi:10.3389/fpls.2018.01882)

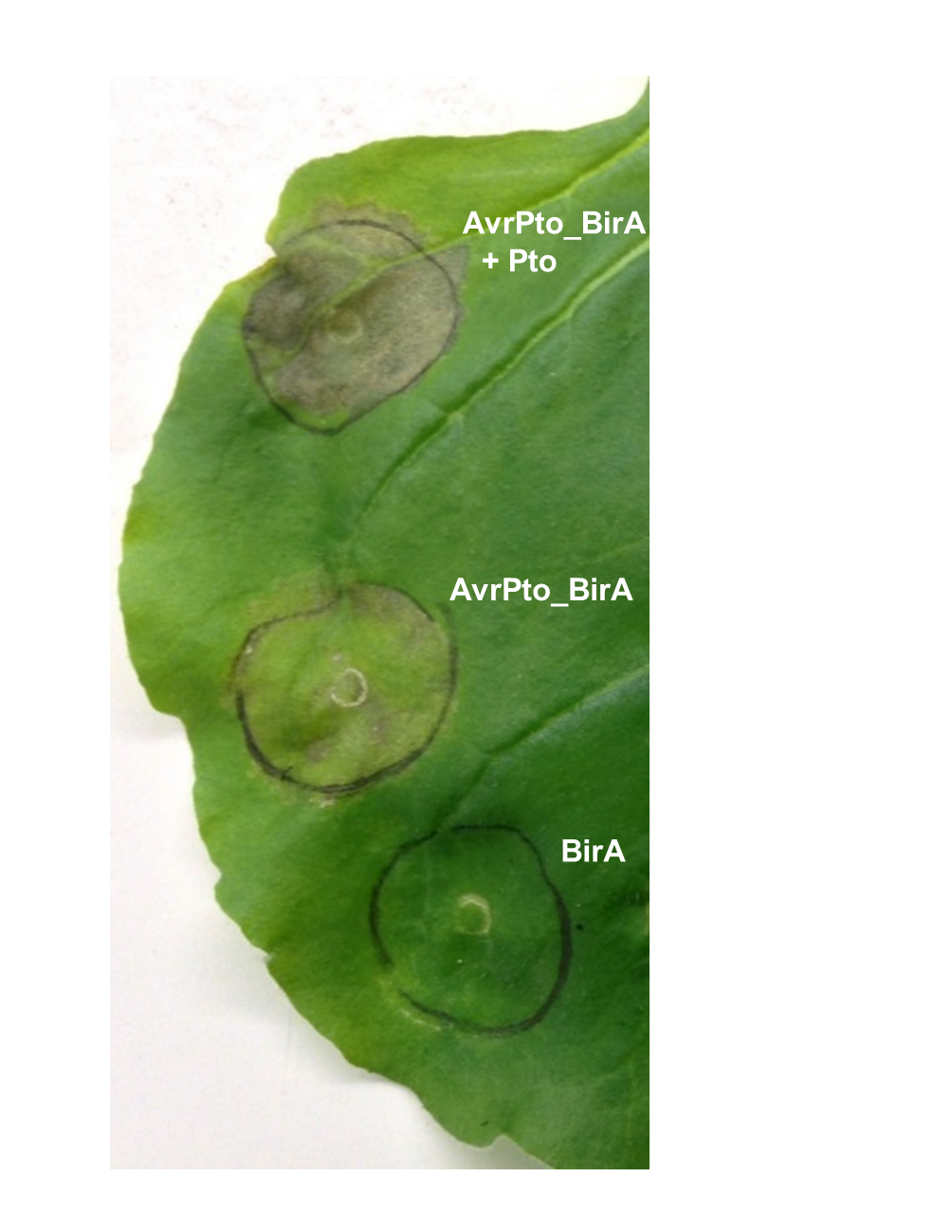

Supplement: FIGURE S1 — Expression of AvrPto_BirA fusion in N. benthamiana. Transient expression of AvrPto_BirA in N. benthamiana revealed that the protein produces a strong hypersensitive response when expressed with Pto. The photo was taken 5 dpi. [file Image_1.TIF]

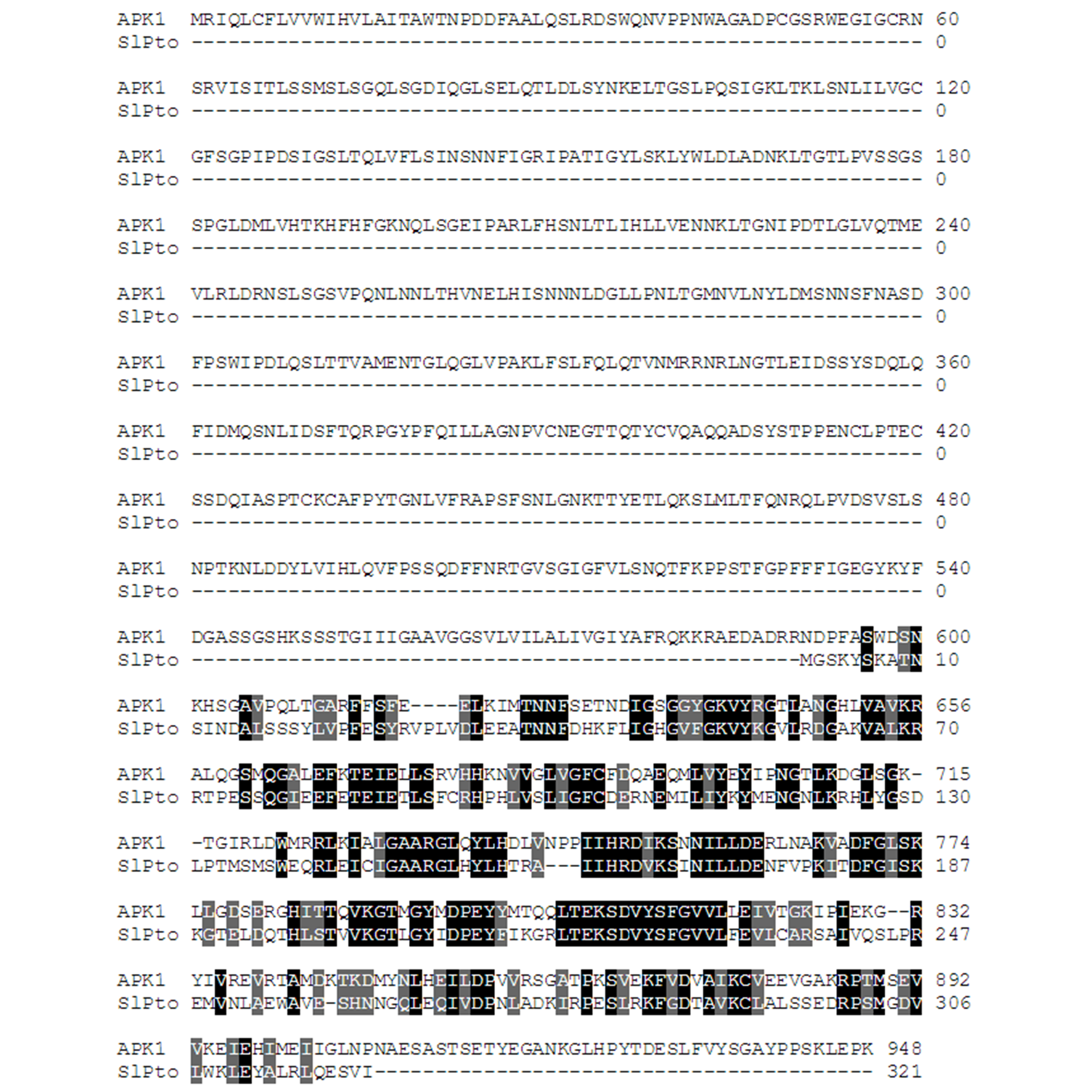

Supplement: FIGURE S2 — Alignment of APK1 and tomato Pto kinase, a known AvrPto interactor. The kinase domains contain 45% amino acid identity and 63% positive identities. [file Image_2.TIF]

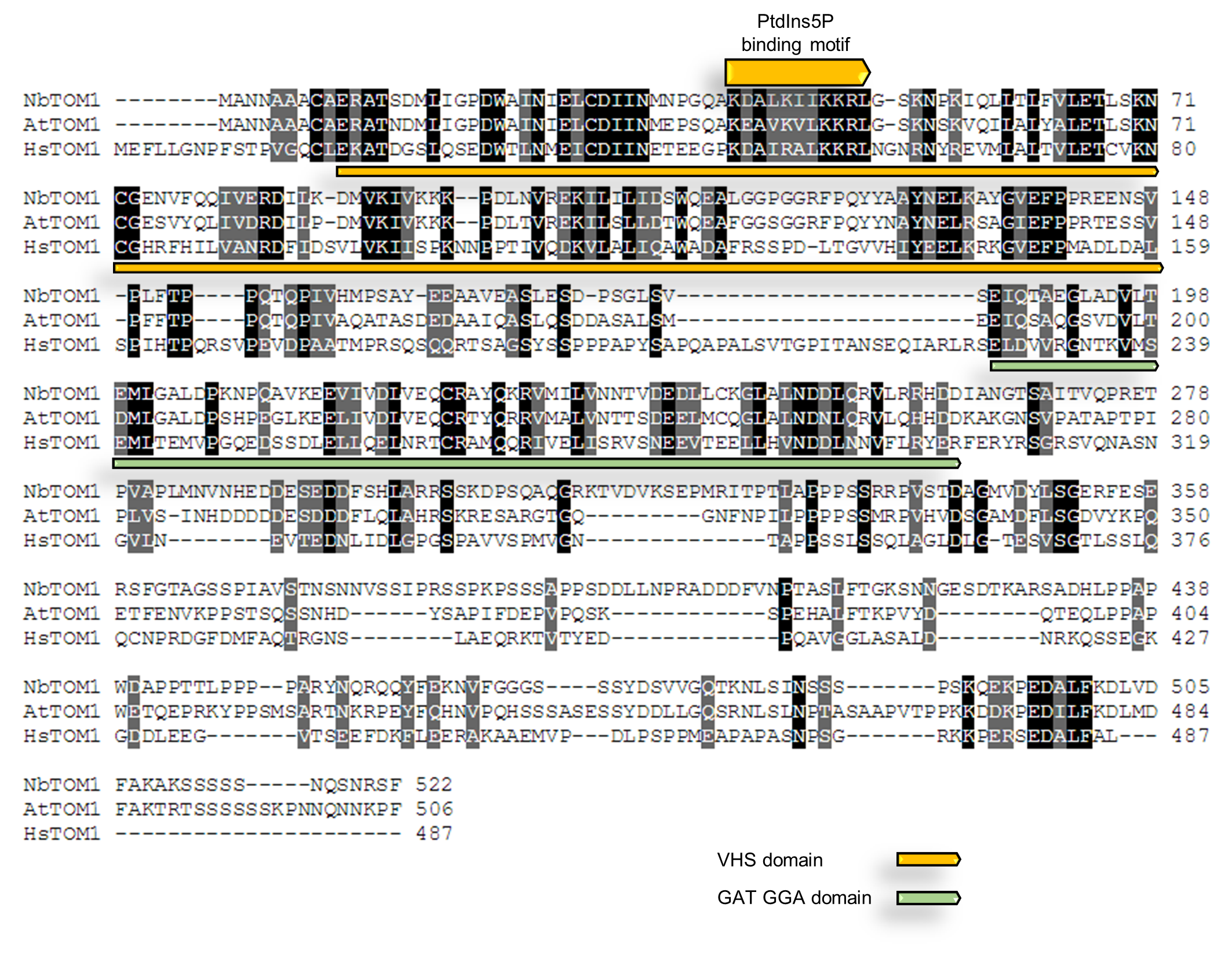

Supplement: FIGURE S3 — Alignment of TOM1 from N. benthamiana, A. thaliana and Homo sapiens. The alignment of the amino acid sequences of TOM1 showing the conservation of the VHS and GAT GGA domains as well as the PtdIns5P binding motif. NbTOM1 (KX272620), AtTOM1 (At1g21380), HsTOM1 (NP_001076437.1). [file Image_3.TIF]

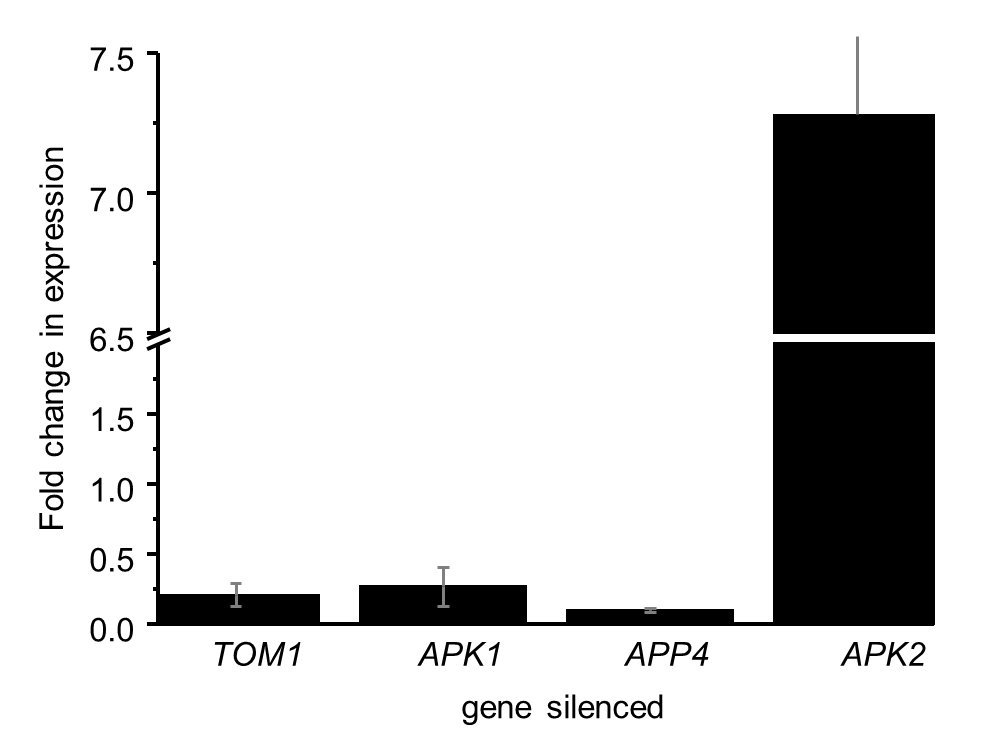

Supplement: FIGURE S4 — Effectiveness of VIGS based gene silencing of N. benthamiana APP genes. Q-RT PCR analysis of APP gene expression in N. benthamiana plants that were silenced for the individual APP genes. [file Image_4.TIF]

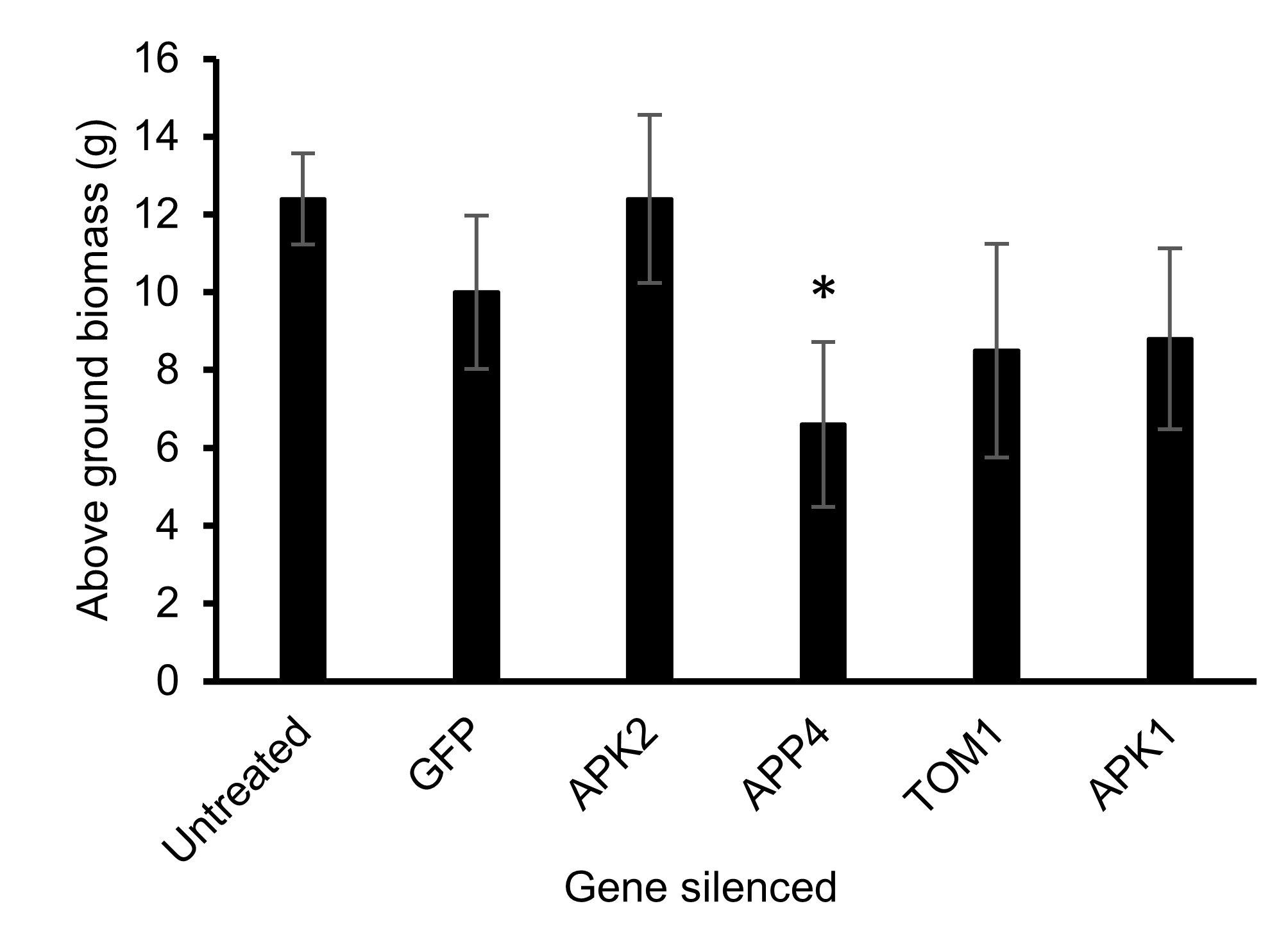

Supplement: FIGURE S5 — Above ground biomass of N. benthamiana plants silenced for APP genes. Plants silenced for the APP genes were harvested at 7 weeks of age and the above ground wet biomass was measured. The ∗ indicates a p-value < 0.05 as compared to untreated wild type plants. [file Image_5.TIF]

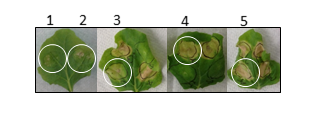

Supplement: FIGURE S6 — Representation of hypersensitive response scoring system. The HR score ranged from 1 which is the beginning of the herpsensitive response showing a discernible collapse of the tissue and a shiny appearance, to 5 which is complete necrosis with the leaf tissue appearing dried and dead. [file Image_6.TIF]
